# Dynamics of the bacterial gut microbiota during controlled human infection with *Necator americanus* larvae

**DOI:** 10.1080/19490976.2020.1840764

**Published:** 2020-11-23

**Authors:** Q. R. Ducarmon, M. A. Hoogerwerf, J. J. Janse, A. R. Geelen, J. P. R. Koopman, R. D. Zwittink, J. J. Goeman, E. J. Kuijper, M. Roestenberg

**Affiliations:** a Center for Microbiome Analyses and Therapeutics, Leiden University Medical Center, Leiden, The Netherlands; b Department of Medical Microbiology, Leiden University Medical Center, Leiden, The Netherlands; c Department of Parasitology, Leiden University Medical Center, Leiden, The Netherlands; d Department of Biomedical Data Sciences, Leiden University Medical Center, Leiden, Netherlands; e Department of Infectious Diseases, Leiden University Medical Center, Leiden, The Netherlands

**Keywords:** Gut microbiota, helminth, hookworm, *Necator americanus*, controlled human infection, longitudinal

## Abstract

Hookworms are soil-transmitted helminths that use immune-evasive strategies to persist in the human duodenum where they are responsible for anemia and protein loss. Given their location and immune regulatory effects, hookworms likely impact the bacterial microbiota. However, microbiota studies struggle to deconvolute the effect of hookworms from confounders such as coinfections and malnutrition. We thus used an experimental human hookworm infection model to explore temporal changes in the gut microbiota before and during hookworm infection. Volunteers were dermally exposed to cumulative dosages of 50, 100 or 150 L3 *Necator americanus* larvae. Fecal samples were collected for microbiota profiling through 16S rRNA gene amplicon sequencing at weeks zero, four, eight, fourteen and twenty. During the acute infection phase (trial week zero to eight) no changes in bacterial diversity were detected. During the established infection phase (trial week eight to twenty), bacterial richness (Chao1, *p* = .0174) increased significantly over all volunteers. No relation was found between larval dosage and diversity, stability or relative abundance of individual bacterial taxa. GI symptoms were associated with an unstable microbiota during the first eight weeks and rapid recovery at week twenty. *Barnesiella*, amongst other taxa, was more abundant in volunteers with more GI symptoms throughout the study. In conclusion, this study showed that clinical GI symptoms following *N. americanus* infection are associated with temporary microbiota instability and relative abundance of specific bacterial taxa. These results suggest a possible role of hookworm-induced enteritis on microbiota stability.

## Introduction

Helminths such as hookworms can have beneficial effects on auto-immune diseases^1,2^ such as celiac disease,^3,4^ but also cause eosinophilic gastroenteritis, anemia and protein loss and are therefore responsible for a high burden of disease in low- and middle-income countries.^5^ As a part of the human gut microbiome in developing countries with a high rate of hookworm infections, hookworms can exert evolutionary pressure on the bacterial gut ecosystem through intestinal motility, mucin glycosylation, mucus secretion, epithelial damage and worm products.^6^ For example, several helminths and their products have been shown to increase permeability of monolayers in cell culture.^7,8^ In addition, worm products can have direct antibacterial activity, thereby having the potential to directly alter the bacterial gut microbiota.^9,10^ However, the complex interplay between hookworms such as *Necator americanus* and the bacterial microbiota is largely unknown.

In real-world settings, most studies have focused on characterizing the gut microbiota of infected individuals in highly endemic regions with limited follow-up on individuals.^11,12^ However, effects of confounding factors cannot always be uncoupled from the bacteria-helminth relationship, as mixed helminth infections, other intestinal diseases and malnutrition are also common in endemic regions.^13^ These factors may explain a large part of inconsistent findings between studies.^13^ In addition, due to the high inter-individual variability of the microbiome, cross-sectional studies only yield limited information.

In the current study, we studied the effect of hookworm infection on the gut microbiota using a longitudinal model for human hookworm infection in healthy volunteers (controlled human hookworm infection model, CHHIM). Here, samples can be obtained at baseline, where the gut microbiome is unperturbed, and longitudinally in order to model the ecosystem’s dynamics and perturbation after exposure to *N. americanus*. This model allows for studying the changes in the bacterial microbiota in the different stages of infection; skin penetration, (pulmonary) migration and gut establishment, a process which takes roughly four weeks.^14^ In addition, potential confounding factors which could affect the outcome of studies investigating bacterial-helminth interactions are minimized.^13^ The power of CHHIM to investigate changes in the human microbiota has been demonstrated in a small study where patients with celiac disease were experimentally infected.^15–17^ Although a very small study (n = 8), a minor increase in richness was seen after infection, while no changes in community, diversity or abundance of individual taxa occurred.^15^ This study was however limited by the use of a low infectious inoculum of twenty larvae which resulted in egg output much lower than commonly found in endemic areas and by only including patients with celiac disease.^18^ In this study we infected individuals with 50–150 L3 larvae, after which we found mean egg counts of around 1500 eggs per gram feces at plateau level, which is more in line with the endemic situation where mild infection is defined by WHO as <2000 eggs per gram feces.^18,19^ Still, infection levels in CHHIMs are not fully comparable to areas with a high infectious burden, defined as >4000 eggs per gram by WHO. The current study had two main aims. First, to investigate temporal changes in the gut microbiota in response to different dosages (ranging from 50 to 150L3) *N. americanus* larvae in healthy young volunteers. Second, to investigate temporal differences in the gut microbiota between healthy volunteers experiencing different amounts of clinical symptoms.

## Results

Results of the clinical trial have been published elsewhere.^19^ Briefly, of the 24 randomized volunteers, twenty completed follow-up and were included in the microbiota analysis, providing a total of 100 fecal samples. The primary aim of the clinical trial was to investigate the effect of repeated infectious dosages on hookworm egg excretion and variability. From our 20 volunteers, eight (40%) were male and twelve (60%) were female and the mean age was 25.7 years (standard deviation 6.1 years). No volunteers had used antibiotics in the six weeks prior to enrollment. All volunteers developed patent hookworm infection as shown by positive microscopy for hookworm eggs at a median of eight weeks (range five-nine) after first skin infection with L3 larvae.^19^ Abdominal adverse events in many volunteers starting at three to four weeks after infection were paralleled by eosinophil increases which likely marked the timepoint of arrival and establishment of the hookworm in the duodenum. Abdominal adverse events consisted of bloating, nausea, vomiting, diarrhea or abdominal cramping. Volunteers exposed to higher larval dosages (n = 6 volunteers with 50L3 larvae, n = 7 with 100L3 larvae and n = 7 with 150L3 larvae) generally had higher egg loads in feces, but there was no relation between cumulative larval dosage and number and severity of adverse events.^19^ Based on the severity, number and duration of adverse events, nine volunteers were classified into the “hi” GI symptoms group, whereas eleven were categorized into the “lo” GI symptoms group by two independent physicians. All volunteers with severe adverse events were placed in the “hi” category, together with two volunteers who did not have severe adverse events but moderate adverse events of long duration (Table S1). Median number of related abdominal adverse events was 4 in the whole cohort (range 0–10), split per dosage group this was 4.5 in the 50L3 group, 4 in the 100L3 group and 3 in the 150L3 group. This difference was not statistically significant. Originally, twelve volunteers were classified in the “hi” category, however, due to severe abdominal adverse events three participants from the “hi” group were treated early and could not be included in the microbiota analysis.

On average 28,600 reads (range = 6,524–49,476 reads, median 29,244 reads) were generated per volunteer sample (total n = 100), resulting in a total of 1,258 unique OTUs (after filtering on 0.005% abundance). Both positive controls were highly similar to theoretical expectations, with the DNA standard (n = 2) being more similar to theoretical expectation than DNA extraction controls (n = 3) based on Bray-Curtis distances (Fig S1A + B). Two out of three negative extraction controls did not contain any reads post-filtering and one negative control contained only five reads in total.

### High individual-specific clustering despite N. americanus infection

**Figure 1.**
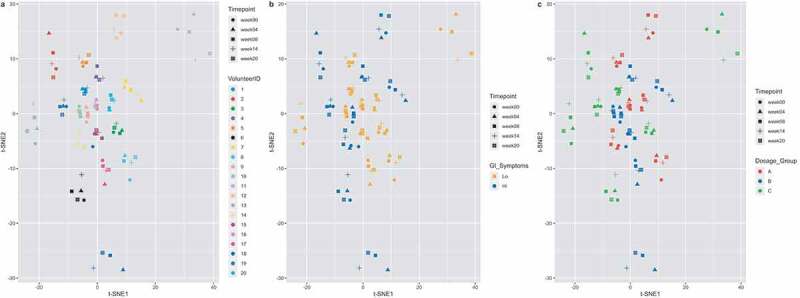
T-Distributed Stochastic Neighbor Embedding (t-SNE), using Bray-Curtis dissimilarity. Volunteer-specific clustering is observed, with no obvious shift according to timepoint. Volunteers (n = 20) are colored by their volunteer number (a), the GI symptoms group (b) or the larval dosage group (c), while each shape corresponds to a timepoint

To explore data and understand potential shifts in microbiota composition, we performed t-Distributed Stochastic Neighbor Embedding (t-SNE), using Bray-Curtis dissimilarity of all samples, which revealed individual-specific clustering (Figure 1a), but no clear clustering according to GI symptoms group (Figure 1b) or larval dosage group (Figure 1c). Two individuals clustered separately, one of which had taken a course of amoxicillin (volunteer 18), while the other was strictly vegetarian (volunteer 11). It needs to be taken into account that t-SNE preserves the local structure rather than the global structure of the data (like in PCA), so large distances in the 2D plot do not necessarily reflect large distances in the high-dimensional space. Other people taking antibiotics during the study course, all for reasons unrelated to the study, did not show large compositional changes (Figure 1 + Fig S2 + Table 1).

**Table 1.** Volunteer characteristics. Included information is larval dosage group, GI symptom group and whether individuals took antibiotics during the study

VolunteerID	Dosage_Group	GI_symptoms	Gender	Antibiotic use		
1	C	Lo	Male	Amoxicilin, three times/day 500 mg, for five days,		
				between trial week zero and four.		
2	C	Hi	Female			
3	C	Lo	Male			
4	B	Hi	Female			
5	A	Lo	Female			
6	C	Hi	Female			
7	B	Hi	Female			
8	A	Lo	Female			
9	B	Hi	Male	Single cefazolin administration, between		
				trial week fourteen and twenty.		
10	B	Lo	Male			
11	C	Lo	Male			
12	A	Hi	Male			
13	C	Lo	Male			
14	A	Hi	Female	Single azithromycin (1000 mg), between		
				trial week four and eight.		
15	B	Lo	Female			
16	A	Lo	Female	Amoxicilin, three times/day 500 mg, for five days,		
				between trial week zero and four.		
17	B	Lo	Male			
18	B	Hi	Female	Amoxicilin, three times/day 500 mg, for five days,		
				between trial week zero and four.		
19	C	Hi	Female			
20	A	Lo	Female

### Larval dosage does not differentially impact alpha diversity or stability in the acute phase of infection

**Figure 2.**
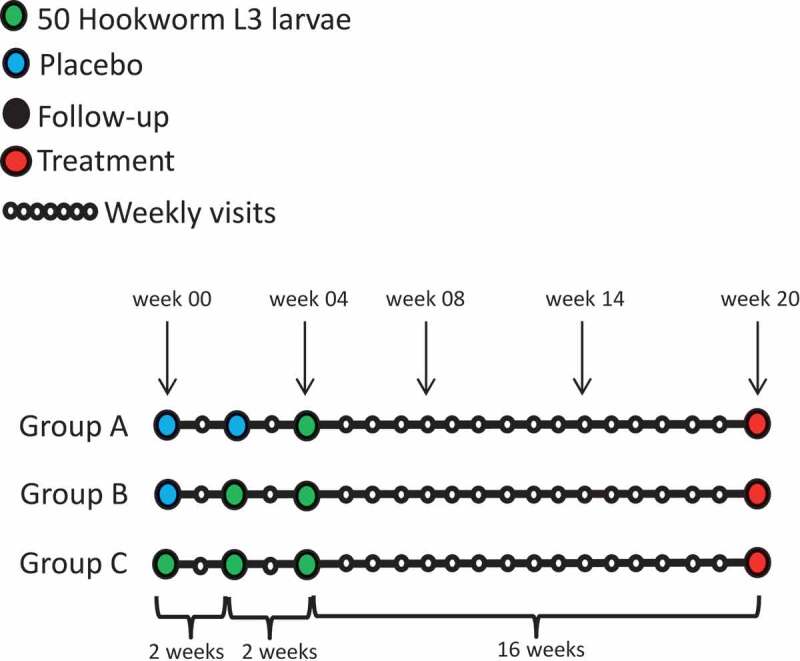
Study design. At indicated trial weeks (week zero, four, eight, fourteen and twenty) feces were collected for microbiota analysis

To investigate whether larval dosages induce differential effects on the gut microbiota, we compared alpha diversity and stability measures between dosage groups. Group A (n = 6 volunteers) received 50L3 larvae, group B (n = 7 volunteers) 100L3 larvae and group C (n = 7 volunteers) 150L3 larvae (Figure 2). First, we investigated potential changes in alpha diversity and stability during the acute phase of infection (which includes trial week zero, four and eight). To test for differences in these parameters, normality was tested using Shapiro-Wilk test and equal variance using an F-test. Subsequently, depending on outcome of these tests, appropriate tests were performed.

**Figure 3.**
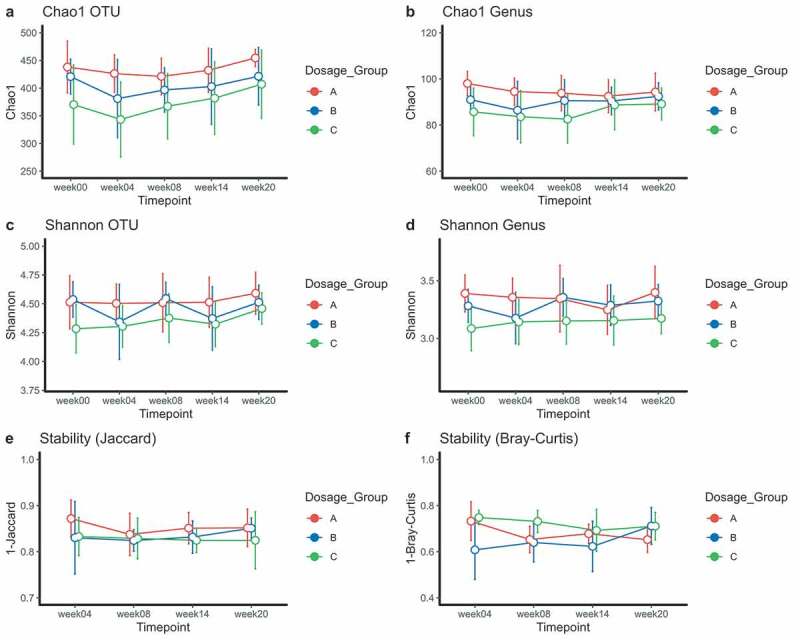
Richness (Chao1) and diversity plots at OTU and genus level (a-d) and stability measures (Jaccard and Bray-Curtis) for larval dosage groups (e-f). Total infectious dosages for group A (red): 50L3 larvae, group B (blue): 100L3 larvae and C (green): 150L3 larvae. Means and the 95% CI of the standard error of the mean are displayed. *p < .05, **p < .01, ***p < .001

We started to compare the effect of acute infection compared to an uninfected state. As at trial week four group A was not yet exposed, and group C twice (Figure 2), we compared their deltas at trial week four (Chao1/Shannon at week four minus Chao1/Shannon at week zero). Group B was not included in this analysis, since this group was infected at week two of the study and thus at the time samples were taken (week four), patent infection was not yet established in the gut. No differences in deltas were found at OTU level (independent t-test, *p* = .76) or genus level (Mann-Whitney test, *p* = .61) between A and C. No difference within group C between trial week zero and four at OTU level (paired t-test, *p* = .49) or genus level (paired t-test, *p* = .41) was observed either (Figure 3a + B). The same tests were performed for Shannon diversity, stability measures (1-Bray-Curtis and 1-Jaccard, Welch t-test *p* = .742 and independent t-test *p* = .219 respectively), but no differences were observed (Figure 3c-f).

At trial week eight all volunteers likely had established intestinal hookworm infection. Therefore, trial week zero and eight were compared to all individuals. No differences were observed in Chao1 at OTU level (Wilcoxon signed rank test, *p* = .391) nor at genus level (Wilcoxon signed rank test, *p* = .152) (Figure 3a + B). No differences were observed in Shannon diversity either (Figure 3c + D).

In conclusion, we did not observe any changes in diversity or stability of the microbiota during the acute phase of infection between or within dosage groups.

### Microbiota richness increases in all volunteers during the established infection phase

Subsequently, we investigated the effect of established infection (trial week eight to twenty) on the gut microbiota using a linear mixed model (LMM). Chao1 at OTU level increased from trial week eight to twenty (*p* = .0174), and less clearly so at genus level (*p* = .0905) over all volunteers, but no differential effect between larval dosage groups was observed (Figure 3). No differences in Shannon diversity or stability were seen between or within larval dosage groups or over time across all individuals. In conclusion, we found an increased richness over all volunteers during established infection, but Shannon diversity and stability remained unchanged. It is however unclear whether this increased richness is a direct result of the infection, as no non-infected group was available at this time point.

### Individual bacterial taxa do not display major differential changes between larval dosage groups

Lastly, we performed differential abundance analysis between larval dosage groups A and C over time using the MetaLonDA package and investigated an overall hookworm effect over all volunteers using DESeq2. Group B was not included in this analysis, as data were only available two weeks after infection. At this timepoint no patent infection is established in the gut, but systemic effects or effects of early larval migration cannot be excluded. In addition, the antibiotic-induced effect on the gut microbiota of volunteer 18 (who was in group B) could affect the analysis, especially considering the small number of volunteers in each dosage group. MetaLonDA at genus revealed that *Dorea* was significantly increased between trial week four and week eight in group A (*p* = .04) (Fig S3A). However, as this is the only differentially abundant taxon at a single time interval, this is unlikely to represent biological relevance. This analysis was also performed at OTU level (Table S2 and Fig S4A). No differences in relative abundance were observed across all volunteers from trial week zero to twenty either at both genus and OTU level (adj. *p*-value > 0.05). We subsequently continued analyzing the relationship between the gut microbiota and severity of GI symptoms.

### Hi GI symptoms were associated with an unstable microbiota at trial week eight

**Figure 4.**
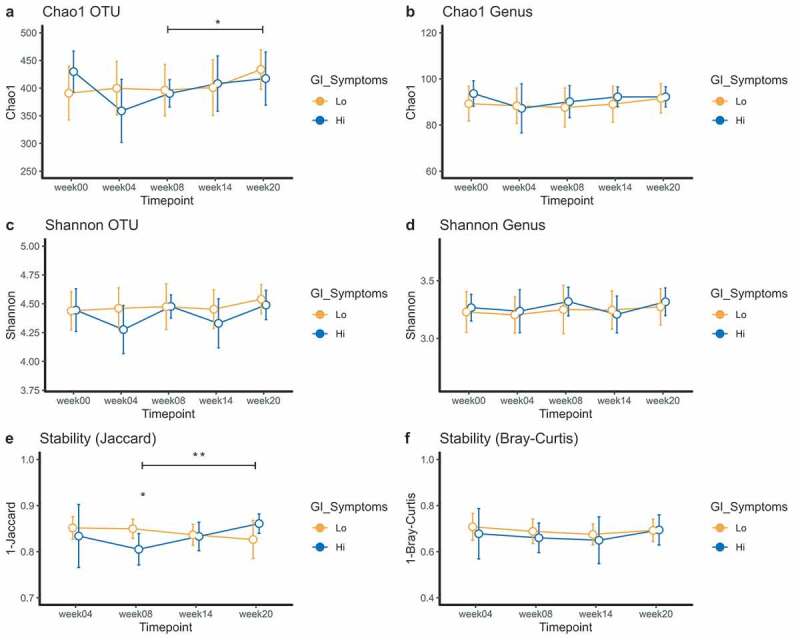
Richness (Chao1) and diversity plots at OTU and genus level (a-d) and stability measures (Jaccard and Bray-Curtis) for GI symptoms groups (e-f). For figure A, significance between trial week eight and week twenty is for the “lo” GI symptoms group (orange). For Figure E, significance between trial week eight and week twenty is for the “hi” GI symptoms group (blue). Means and the 95% CI of the standard error of the mean are displayed. *p < .05, **p < .01, ***p < .001

Our next goal was to investigate whether baseline differences in microbiota composition could be associated with severity of GI symptoms, so we compared the “lo” (n = 11 volunteers) and “hi” (n = 9 volunteers) GI symptoms groups. No difference in Chao1 was observed at OTU level (independent t-test, *p* = .244) or genus level (Mann-Whitney test, *p* = .446) (Figure 4a + B) at week zero. Comparing week zero with week eight did not show differences at OTU level (Wilcoxon signed rank test for the “lo” group, *p* = .391 and paired t-test for “hi” group, *p* = .382, Figure 4a) or genus level (Wilcoxon-signed rank test for “lo”, *p* = .152 and paired t-test for “hi”, *p* = .132) (Figure 4b). No differences were seen at trial week eight between symptom groups at OTU level (Mann-Whitney test, *p* = .412) or genus level (independent t-test, *p* = .674) (Figure 4a + B). The same tests were performed for Shannon diversity, but no differences were observed either (Figure 4c + D).

Microbiota stability of the ”hi” GI symptoms group was significantly decreased at trial week eight as compared to the “lo” GI symptoms group (Jaccard, independent t-test, *p* = .036) (Figure 4e). No difference was found using Bray-Curtis dissimilarity (Figure 4f).

In conclusion, we did not find any changes in alpha diversity between GI symptoms groups, but microbiota stability (here Jaccard) was significantly reduced in the “hi” GI symptoms group at trial week eight compared to the “lo” GI symptoms group.

### Microbiota stability recovers over time

LMM was applied to investigate changes in diversity during the established phase in the GI symptoms groups. We found a significant increase in Chao1 from trial week eight to twenty in the “lo” GI complaints group at OTU level (LMM, *p* = .045) (Figure 4a), but not at genus level (LMM, *p* = .120) (Figure 4b). No differences were found for Shannon diversity.

As previously mentioned, stability in the “hi” GI symptom group was reduced at trial week eight. This instability quickly recovered from trial week eight to twenty (Jaccard, paired t-test, *p* = .004) (Figure 4e). In addition, the slopes between the symptom groups were significantly different from trial week eight to twenty, confirming the recovery within the ”hi” symptoms group (LMM interaction, *p* = .002). This also means that there was increased dissimilarity in the “lo” symptoms group in this time period. We further hypothesized that this stability may perhaps be related to eosinophil count, but we did not find a significant correlation between eosinophil count and microbiota stability when stratifying by trial week (Fig S5A). No differences in Bray-Curtis dissimilarity were observed during established infection, nor was a correlation found with eosinophil counts at any trial week (Fig S5B). Eosinophil counts are visualized per time point in Figure S6.

In summary, the “hi” GI symptom group was characterized by transient microbiota instability and subsequent recovery.

### Specific bacterial taxa differ between symptoms groups during the entire study course

**Figure 5.**
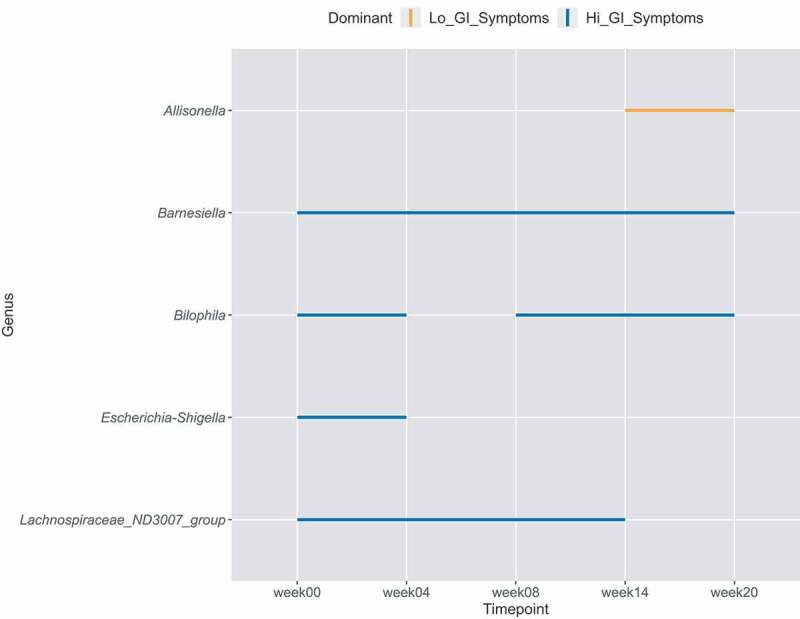
Time intervals of significantly different bacterial genera between GI symptoms groups. Each line interval represents a significant time interval, with significance being considered *p* < .05. Orange lines indicate higher abundance in the “lo” GI symptoms group, while blue indicates higher abundance in the “hi” GI symptoms group

To investigate whether changes in individual bacterial taxa over the entire study course could be linked to symptom groups (‘hi’ n = 9, ‘lo’ n = 11), we employed the MetaLonDa package (Figure 5, Fig S3B-F and Table S2) and found several bacterial taxa in the “hi” GI symptoms group significantly increased at genus level. *Barnesiella* was found to be significantly increased in this group at all intervals from trial week zero to week twenty (*p* <.05), *Lachnospiraceae_ND_3007* was significantly higher from trial week zero to fourteen (*p* <.05), *Bilophila* was more abundant between trial week zero and four, and between trial week eight and twenty (*p* <.05) and *Escherichia-Shigella* was significantly more abundant between trial week zero and four (*p* <.05). In the “lo” GI symptoms group, *Allisonella* was more abundant between trial week fourteen and twenty, at which time a chronic infection had been established (*p* <.05). Lastly, relative abundances over time of these significantly different genera were visualized, to investigate whether significance was driven at the group level or by a single individual (Fig S3B-F). This showed that the difference in *Escherichia-Shigella* was driven by a single person, namely volunteer 18, while all other differences were largely group-driven. When analysis was repeated without volunteer 18, *Escherichia-Shigella* was indeed non-significant (*p* = .292). In addition, the association with *Barnesiella* and *Bilophila* persisted throughout the study (*p* = .074 and *p* = .072), *Allisonella* became more abundant in the “lo” group throughout the entire study (*p* = .017) and *Oscillibacter* was more abundant in the “lo” group (*p* = .012) from week zero to fourteen (Table S2). All analyses were also performed at OTU level (Fig S4B+C and Table S2). These results confirm that differences in relative abundance of taxa between the symptom groups were largely group-driven, apart from *Escherichia-Shigella*.

## Discussion

Herewith we present the first longitudinal assessment of microbiota changes over the course of an experimental *N. americanus* infection in healthy individuals. Although no convincing relationship between microbiota and larval dosage was observed, stability of the bacterial microbiota was linked to severity of clinical symptoms. In addition, we found several statistically significant changes in relative abundance of individual bacterial taxa over time between symptom groups.

We found a very strong volunteer-specific clustering, despite a patent hookworm infection. This corroborates previous findings showing that the gut microbiota is stable over time in healthy adults at a compositional level^20–22^ and the previous assessment of experimental hookworm infections in patients with celiac disease where minor changes were detected over time.^16^

We detected an interesting link between microbiota stability from trial week eight to twenty and clinical GI symptoms. Recovery of stability in the “hi” symptoms groups leads us to believe that either volunteers with a more unstable microbiota in early weeks post-infection are more likely to experience GI symptoms during the infection, or the GI symptoms are caused by a more severe enteritis that also affects microbiota stability. The latter hypothesis seems most likely whereby symptoms are caused by an eosinophilic enteritis, with eosinophils having been described to correlate with severity of enteritis,^19,23^ and the enteritis may in turn affect the gut microbiota. Although cause and effect cannot be determined, it does suggest an important bacterial-helminth-host interplay, which deserves further investigation.

We observed increased richness over all volunteers during the established infection phase. This is in line with previous studies which have mostly shown that individuals with a parasitic infection show either equal or increased microbial richness and diversity.^11,15,24^ However, we cannot be fully certain this is an infection-induced effect, as no “no-infection” control group was included in our study. It is unclear why alpha diversity may increase during hookworm infection, although several hypotheses can be formulated. Potentially, the immune regulatory effects of helminths might facilitate increased bacterial microbiota richness and diversity.^25,26^ On the other hand, expansion of the current bacterial community could also be promoted. Another hypothesis is that *N. americanus* affects the gut metabolome. While the effect of *N. americanus* infection on the full gut metabolome has not yet been investigated in humans to our knowledge, short chain fatty acid (SCFA) levels were measured in eight volunteers undergoing *N. americanus* infection.^27^ Out of these eight volunteers, six showed an increase in total fecal SCFA, while two showed a reduction.^27^ Even though this suggests an effect of *N. americanus* on the gut metabolome, this should be confirmed with a larger sample size.

We observed several changes in individual bacteria taxa between the GI symptoms groups, although the biological relevance of these changes remains unclear. The increased abundance of *Barnesiella* and the decreased abundance of *Allisonella*, a histidine-consuming and histamine-producing taxon, in the “hi” symptoms group are puzzling. While *Barnesiella* is associated with a healthy microbiota and beneficial intestinal effects,^28–31^ *Allisonella* and its metabolic product histamine are associated with increased GI symptoms.^32–34^ This would counterintuitively suggest that individuals with a microbiota generally regarded as more beneficial respond more heavily to hookworm invasion. The opposite holds true however for *Bilophila*, a taxon which thrives under high-fat and animal-based diets.^35,36^ It is associated with increased inflammation, impaired intestinal barrier function and production of hydrogen sulfide.^35–37^ Being more abundant in the “hi” symptom group, this contradicts with the hypothesis that a more beneficial microbiota responds more vigorously to hookworm invasion. All in all, the relevance of these findings should be tested in larger groups and with more functional techniques than 16S rRNA gene amplicon sequencing. In addition, answering this hypothesis would require a clear definition of a ‘healthy or beneficial’ microbiota, a phenomenon which is currently incompletely understood.

The current study had several strengths and limitations. During the study period, five volunteers were prescribed antibiotics. While a clear effect of antibiotic usage was only observed in volunteer 18, we cannot exclude that an effect occurred in other volunteers and might have confounded our results. However, given the small sample size we were unable to partial out any confounders (e.g. antibiotic use and diet) which is a limitation of our study. It should be noted that investigating fecal material is probably not reflective for local microbiota changes in the duodenum and ideally duodenal biopsies would be taken, as was done previously by Giacomin *et al*.^17^ This would however pose a sharply increased burden to volunteers. By using healthy volunteers, the effects of *N. americanus* infection on the bacterial gut microbiota could be studied without many external confounders. In addition, the longitudinal study setup allowed us to investigate the dynamics of the bacterial gut ecosystem. Implementation of well-controlled longitudinal studies was also recently described to be crucial for advancing the microbiome field.^38,39^ Future studies should include functional approaches (e.g. coupling metagenomics with metabolomics) to obtain insight into potential changes in microbial metabolism which could be a result of *N. americanus* infection. By using positive and negative controls, we confirmed that we should investigate richness and diversity both at OTU and genus level, and that our DNA extractions were well performed, although minimal contamination may have occurred. The original clinical trial reported the highest egg counts described in CHHI experiments in literature yet, reaching egg counts similar to those seen in mild infection in endemic areas, allowing for better comparison with natural infection.^18^ Although the groups in this controlled study were small, the combination of infecting healthy volunteers with the highest infectious as of yet and describing their bacterial gut microbiota longitudinally is unique.

In conclusion, this is the first study to investigate longitudinal changes in gut microbiota during *N. americanus* infection in healthy individuals. We observed high stability of the gut microbiota despite this infection over the twenty-week study period, although transient instability was observed in individuals with “hi” GI symptoms. These data open new avenues for exploring helminth-bacterial interaction in the human intestine.

## Materials and methods

Twenty-four healthy male and female volunteers aged 18–45 years were included in a randomized controlled clinical trial investigating the effect of repeated infectious dosages on hookworm egg excretion and variability as previously described.^19^ Volunteers were dermally exposed with two week intervals to either one, two or three dosages of 50 infectious larvae, resulting in cumulative dosages of 50, 100 and 150 larvae respectively. Study set-up was such that the 150 larvae group (C) received first infection at trial week zero, the 100 larvae group (B) at trial week two and the 50 larvae group (A) at trial week four. Volunteers were allocated equally to one of three groups at random according to an independently prepared randomization list. Group allocation was defined by the randomization number which was linked to the volunteer identification code at the first infection. Investigators and participants were blinded to group allocation. A schematic overview of study setup can be found in Figure 2.

Culture of larvae and procedure of infection was performed according to a previously described method.^40^ In short, infective L3 larvae were cultured from feces from a chronic donor, were suspended in water and applied on upper arms and calves using gauzes. Volunteers were followed for twenty weeks after first exposure, after which treatment with albendazole was given to eradicate the infection. Volunteers visited the trial center weekly for collection of adverse events, safety laboratory evaluation and collection of fecal samples for egg count. Adverse events were collected at weekly visits. For every adverse event, time and date of onset, end, severity and causality were recorded. Adverse events were characterized using ICD-10 as unrelated, unlikely, possibly, probably or definitely related to hookworm infection, and mild (no interference with daily life), moderate (discomfort interfering with daily life) or severe (causing inability to perform usual daily activity). Adverse events were then assessed by two independent physicians who divided the participants in two groups with ‘hi’ and ‘lo’ adverse events. Originally, twelve volunteers were classified in the “hi” category. All volunteers with severe adverse events were placed in the “hi” category, together with two volunteers who did not have severe adverse events but moderate adverse events of long duration (Table S1). Consensus was reached for every participant. Unfortunately three participants in the “hi” group withdrew early from the trial due to severe abdominal adverse events and insufficient follow-up fecal samples were collected to include these participants in the microbiome analysis. For analysis, adverse events scored as possibly, probably and definitively related were considered related and were included in the symptom grouping. Duration of adverse events was recorded. Detailed information on adverse events for each volunteer can be found in Table S1. Samples for analysis of fecal microbiota were collected at baseline and trial weeks four, eight, fourteen and twenty of the trial. For analysis of the relation between clinical symptoms and fecal microbiota, gastrointestinal symptoms were categorized as either “hi” or “lo”.^19^Fecal egg counts were measured by microscopy using Kato-Katz. Eosinophils were measured weekly and egg counts were measured weekly from trial week five using Kato Katz microscopy. The trial was approved by the LUMC IRB (P17.224) and was registered at clinicaltrials.gov under NCT03257072.

### Microbiota analysis

Fecal samples were aliquoted and immediately stored at −80 C. DNA was extracted from 0.1 gram feces using the Quick-DNA™ Fecal/Soil Microbe Miniprep Kit (ZymoResearch, CA, USA) according to manufacturer instructions with minor adaptations, as described previously.^41^ Quality control, library preparation and sequencing were performed by GenomeScan B.V. (Leiden, The Netherlands) using the NEXTflex™ 16S V4 Amplicon-Seq Kit (BiooScientific, TX, USA) and the Illumina NovaSeq6000 platform (paired-end, 150bp). Raw read processing was performed using the NG-Tax 0.4 pipeline with following settings: forward read length of 120, reverse read length of 120, ratio OTU abundance of 2.0, classify ratio of 0.9, minimum threshold of 1*10^−7^, identity level of 100% and error correction of 98.5, using the Silva_132_SSU Ref database.^41–43^ The obtained OTU table was filtered for OTUs with less than 0.005% relative abundance.^44^ As quality controls for both DNA extraction and sequencing, we included ZymoBiomics Microbial Community Standard (Zymo Research, Irvine, California, USA) ZymoBiomics Microbial Community DNA Standard (Zymo Research) and three negative DNA extraction controls. Raw sequencing data are available at ENA (https://www.ebi.ac.uk/ena) under accession number PRJEB36316. All analytical R code can be found at https://github.com/qducarmon/hookworm_microbiota_16S. .

### Statistical analysis

All analyses were performed in R (v3.6.1) using the packages phyloseq (v1.28.0), microbiome (v1.6.0), Metalonda (v1.1.5), DESeq2 (v1.24.0), lme4 (v1.1–21) and lmerTest (v3.1–0).^45–50^ Richness and diversity were computed at OTU and genus level, as richness was found to be overestimated based at OTU level in the positive controls. Genus level was obtained by agglomerating OTUs at genus level. Bray-Curtis and Jaccard indices were computed at genus level. Bray-Curtis and Jaccard indices were computed intra-individually, using trial week zero as the baseline measurement. As Bray-Curtis and Jaccard indices are dissimilarity indices, we computed 1- respective index to obtain similarity, where a value of 1 represents 100% similarity. For alpha diversity and stability measures, data were split into an “acute infection phase” (trial week zero to eight) when most symptoms occurred and an “established infection phase” (trial week eight to twenty) when symptoms subsided. To test for differences in these parameters, normality was tested using Shapiro-Wilk test and variance was tested using an F-test. Subsequently, depending on outcome of the normality and variance test, independent t-tests, Welch t-tests, paired t-tests, Mann-Whitney U tests and Wilcoxon signed-rank tests were performed. Clustering using t-Distributed Stochastic Neighbor Embedding (t-SNE) method was performed using the tsne_phyloseq function with default parameters.^51^ t-SNE aims to preserve the local structure of the original high-dimensional space while projecting the data points in a low dimensional (2D) space. All figures were created in R and only minimally formatted in Adobe Illustrator when necessary.

#### Correlation analysis

We used Spearman’s Rank correlation to examine the relationship between eosinophil count and microbiota stability. Microbiota stability was defined in the same manner as previously, with Bray-Curtis and Jaccard indices computed intra-individually, using trial week zero as the baseline measurement. As both indices are dissimilarity indices, 1- respective index was computed to obtain similarity. Eosinophil count was measured weekly, and therefore each individual at each time point had a measured eosinophil count. Timepoints were stratified to account for the repeated measurements design. In order to avoid skewing of the correlation by baseline data, at which point eosinophils were low and microbiota was 100% similar due to baseline comparison, this timepoint was excluded.

#### Linear mixed models

We performed linear mixed modeling (LMM) using the lmer function from the lme4 package^49^ for alpha diversity and both stability indices from trial week eight until week twenty, as all groups had established infection in the gut from trial week eight onwards. Volunteer ID was included as a random intercept to control for inter-individual baseline differences and repeated measurements design. Included fixed effects were dosage group/symptom group and timepoints. In case an interaction effect was suspected by visually inspecting plots, an additional interaction model was also performed with dosage group/symptom group*timepoint. Models were checked by inspecting whether residuals were normally distributed using qq-plots. *P*-values were obtained using the lmerTest package and considered significant when <0.05.^50^

#### Time series modeling of individual taxa

Differential abundance testing was performed at genus and OTU level. The metagenomic longitudinal differential abundance method (MetaLonDA) package was used to identify differentially abundant taxa between groups over time.^47^ It is a flexible method capable of handling inconsistencies often observed in human microbiome studies and relies on two main modeling components, the negative binomial distribution for modeling read counts and smoothing spline ANOVA for modeling longitudinal profiles. The function metalondaAll was used with the following settings: n.perm = 1000, fit.method = ”nbinomial”, num.intervals = 4, *pvalue*.threshold = 0.05, adjust.method = ”BH”, norm.method = ”median_ratio”. These settings indicate that the function was run with 1000 permutations using the median ratio method to normalize count data and fitting a negative binomial distribution. Four intervals were included (between each included trial week) and *p*-values were corrected using the Benjamini-Hochberg procedure. DESeq2 was used to establish an overall time effect across all volunteers using the likelihood-ratio-test (full model included volunteer ID and timepoint, reduced model included volunteer ID) and for identifying differentially abundant taxa in pair-wise comparisons.^48^ Prior to the use of both MetaLonDA and DESeq2, genera and OTUs were filtered for presence in at least 25% of all samples. Relevant tests performed are indicated in all figures and in the text.

## Disclosure of Potential Conflicts of Interest

No potential conflicts of interest were disclosed.

## Supplementary Material

Supplemental Material
