# Supplementary material for: Dynamics of the bacterial gut microbiota during controlled human infection with Necator americanus larvae
Source: Gut Microbes. 2020 Nov 23;12(1):1840764. doi: 10.1080/19490976.2020.1840764 (PMC7714523; doi:10.1080/19490976.2020.1840764)
Supplement: Supplemental Material [file KGMI_A_1840764_SM7651.zip › Supplementary information/Supplementary figures legends.docx]

**Supplementary figures**

**Figure S1**: Compositional profiles of positive controls (A) (DNA standard and mock community) and a heatmap based on Bray-Curtis dissimilarity (B) where 0 indicates an identical compositional profile, while higher numbers indicate more dissimilar profiles.

**Figure S2**: Compositional profiles at family level for all volunteers. Other indicates the sum of all bacterial families not specifically indicated in the legend, while Not_Available indicates reads which were not classified. “Hi” and “Lo” indicate the GI symptom groups and the numbers (one to twenty) indicate volunteer ID. * indicates the two time points between which volunteers (if applicable) were prescribed antibiotics.

**Figure S3**: Relative abundances of differentially abundant genera identified by MetaLonDa, *Dorea* (A) *Allisonella* (B), *Barnesiella,* (C), *Bilophila* (D), *Escherichia-Shigella* (E) and *Lachnospiraceae_ND_3007_group* (F). The curves show locally weighted scatterplot smoothing (LOESS) per symptom group (blue for “hi” GI symptoms, orange for “lo” GI symptoms). Shaded area indicates 95% confidence interval.

**Figure S4**: Time intervals of significantly different bacterial OTUs between dosage group A and C (A), GI symptoms groups (B) and GI symptoms groups without volunteer 18 (C). Each line interval represents a significant time interval, with significance being considered p<0.05.

Red lines indicate higher abundance in dosage group A, green lines indicate higher abundance in dosage group C. Orange lines indicate higher abundance in the “lo” GI symptoms group, while blue indicates higher abundance in the “hi” GI symptoms group.

**Figure S5**: Spearman’s Rank correlation plots of eosinophil counts with stability measures stratified by trial week.

**Figure S6**: Dot plot of eosinophil counts (*10^9^/L) of the GI symptoms groups per trial week. Orange dots indicate the “lo” GI symptoms group, blue dots indicate “hi” GI symptoms group.

**Table S1:** Detailed information for each volunteer on the amount, duration and type of adverse events. Volunteers have been ranked by severity of the symptoms, with the volunteer experiencing most symptoms being on top of the table. Coloring indicates severity of adverse events and duration of symptoms (red indicates symptoms for more than 100 days, orange for 50-99 days and yellow for 10-49 days).

**Table S2:** Significantly differentially abundant genera and OTUs between “hi” and “lo” symptom groups and dosage group A and C as identified by MetaLonDA, their corresponding p-values and the time intervals during which they were significant.
